# Expression of *dnaE2* promotes genetic diversity in mycobacterial biofilms

**DOI:** 10.3389/fcimb.2025.1647744

**Published:** 2025-12-01

**Authors:** S. Salini, Sinchana G. Bhat, M. Nijisha, Kumar Paritosh, Reshma Ramachandran, Nikhil Nikolas, Saba Naz, Krishna Kurthkoti

**Affiliations:** 1 Mycobacterium Research Laboratory, Rajiv Gandhi Centre for Biotechnology, Thiruvananthapuram, India; 2 Pathogen Biology Laboratory, Rajiv Gandhi Centre for Biotechnology, Thiruvananthapuram, India; 3 Centre for Genetic Manipulations of Crop Plants, University of Delhi, New Delhi, India; 4 Department of Biology, Indian Institute of Science Education and Research, Tirupati, India; 5 School of Biosciences, Council of Scientifc and Industrial Research (CSIR)-Centre for Cellular and Molecular Biology, Hyderabad, India

**Keywords:** mycobacteria, biofilms, mutagenesis, persistence, fitness defect, bacterial evolution, *mmpL11*

## Abstract

The complex cellular architecture and microenvironments within biofilms give rise to a physiologically and genetically heterogeneous population. Transcriptome analysis of *Mycobacterium smegmatis* from biofilm culture and its transition phase into planktonic growth was performed to identify the genetic basis of heterogeneity in the biofilm. Biofilms displayed redox imbalance, resulting in increased levels of reactive oxygen species and activation of the mycobacterial mutasome consisting of *dnaE2*, *imuA’*, and *imuB*. Whole-genome sequencing of biofilm and planktonic cultures revealed a modest increase in the number of allelic variants in the biofilm culture compared with the planktonic culture. Deletion of *dnaE2* reduced mutation frequency and bacterial fitness compared with the parental strain in biofilm culture and reduced allelic variation in the culture. Our study uncovered the multiple benefits of *dnaE2* expression in biofilms, such as increased genetic diversity and reduced growth rate, both of which are necessary for mycobacterial survival and adaptation.

## Introduction

Bacterial biofilms are complex, sessile bacterial communities with heterogeneous populations that display different growth dynamics and physicochemical properties. Biofilms not only enable bacterial communities to withstand environmental stresses such as nutritional starvation, host defense mechanisms, and antimicrobial agents but also allow them to evolve and cause a resurgence of infection when favorable conditions return (Hall-Stoodley et al., 2004; Hansen et al., 2007; Steenackers et al., 2016). Biofilm formation is initiated by quorum sensing of autoinducer molecules such as acyl-homoserine lactone and nucleotide second messengers (Camilli and Bassler, 2006; Pesavento and Hengge, 2009) triggering a shift in cell fate from a swarmer to a sedentary state. This lifestyle change is followed by the production of an extracellular matrix consisting of polysaccharides, proteins, and extracellular DNA (eDNA) that encapsulates the bacterial community as a single ecosystem (Branda et al., 2005; López et al., 2010; Vlamakis et al., 2013). The simultaneous occurrence of microenvironments, variations in gene expression, and emergence of mutants result in chemical and genetic heterogeneity within the bacterial population of biofilms (Wimpenny et al., 2000; Stewart and Franklin, 2008; Williamson et al., 2012; Kolter et al., 2015; García-Betancur and Lopez, 2019). These differences confer the ability to respond to environmental stress and resist the action of antibiotics (Sadiq et al., 2017). Infections caused by pathogenic bacteria have become a serious public health concern because of their ability to form biofilms both within the body and on medical devices (Donlan, 2001; Schulze et al., 2021).

Members of the *Mycobacterium* genus form biofilms with a pellicle at the air–liquid interface in the absence of media-compatible detergents such as Tween or can be triggered by reductive stress (Ojha et al., 2005; Trivedi et al., 2016). While *in vitro* biofilms of *Mycobacterium* species have proved helpful in understanding bacterial physiology, their relevance in disease remained underappreciated until recently, when Chakraborty et al. demonstrated the formation of biofilms of *Mycobacterium tuberculosis* in infected lung tissues of mice and humans (Chakraborty et al., 2021). During biofilm formation, glycopeptidolipids facilitate mycobacterial attachment (Recht et al., 2000; Recht and Kolter, 2001; Yang et al., 2017), followed by the synthesis of specialized short-chain C_56_–C_68_ free mycolic acids that are necessary for biofilm maturation (Ojha et al., 2005). Transcriptomic analysis of mycobacterial biofilms has shown the importance of the exochelin uptake system and GlnR-regulated genes in iron uptake, nitrogen assimilation, and resistance to peroxide stress (Ojha and Hatfull, 2007; Yang et al., 2018). Biofilms from either fast- or slow-growing *Mycobacterium* species contain populations of antibiotic-tolerant bacteria that can survive high levels of antibiotic treatment (Ojha et al., 2008; Rose et al., 2015; Trivedi et al., 2016), posing a great concern for the outcome of drug therapy. In *M. tuberculosis*, thiol-induced reductive stress accelerates biofilm formation, which harbors drug-tolerant populations (Driffield et al., 2008; Conibear et al., 2009; Ryder et al., 2012; Penterman et al., 2014; Steenackers et al., 2016).

Metabolic changes during biofilm formation have been shown to increase reactive oxygen species production, causing DNA damage. Extensive DNA damage induces the SOS response, which is characterized by cell division arrest and often mutagenesis. *Mycobacterium* has adapted two types of DNA damage responses to genotoxic assaults: (i) the LexA/RecA-dependent SOS pathway and (ii) the LexA/RecA-independent PafBC pathway, a master transcriptional regulator encoded in the Pup proteasome gene locus (Movahedzadeh et al., 1997; Müller et al., 2018; Adefisayo et al., 2021). While the LexA/RecA pathway is triggered following DNA damage, activation of the PafBC system is not directly induced by DNA damage (Adefisayo et al., 2021). Despite these differences, quinolone drugs and replication perturbations can activate the SOS response via the PafBC system. The induction of the SOS response is often associated with increased mutagenesis caused by error-prone DNA polymerases. In *E*. *coli*, during the SOS response, two Y-family polymerases with low fidelity, processivity, and higher error rates—DinB (Pol IV) and UmuDC (Pol V)—are recruited to bypass DNA lesions (Jarosz et al., 2007). Translesion synthesis (TLS) of DNA plays a key role in the evolution of fitness and in conferring antibiotic resistance (McKenzie et al., 2000; Barrett et al., 2019). Until recently, the role of mycobacterial DinB proteins in mutagenesis was not well understood. It has been demonstrated that mycobacterial DinB2 can lead to substitution mutations and promote frameshifts in homopolymeric sequences as well (Dupuy et al., 2023). In *the Mycobacterium* genus, the *E. coli* equivalents of Pol IV and UmuDC are missing; however, the C-family polymerase DnaE2 participates in stress-induced mutagenesis (Boshoff et al., 2003; Ditse et al., 2017). It has been demonstrated that in *M. smegmatis and M. tuberculosis*, during the SOS response, two additional genes, *imuA’* and *imuB*, are induced and required for mutagenesis, constituting the mycobacterial mutasome cassette (*dnaE2*, *imuA’*, and *imuB*) (Warner et al., 2010). Because DnaE2 lacks the crucial β-clamp binding motif, ImuB assists in recruiting the enzyme for repair. In addition, ImuB interacts with ImuA’ through an extended C-terminal region, indicating that ImuB may be a crucial accessory factor in the mutasome (Warner et al., 2010; Gessner et al., 2023).

Although multiple studies have identified the genes required for biofilm formation and maturation, the basis of genetic heterogeneity and mutagenesis in mycobacterial biofilms remains poorly understood. In the present study, we investigated the role of DnaE2 and its accessory factors ImuA*’* and ImuB, which constitute the mycobacterial mutasome (Boshoff et al., 2003; Warner et al., 2010). Transcriptomic and reporter gene analyses showed induction of the mutasome in the biofilm and its reduction during recovery to the planktonic stage. Our findings further reveal that the expression of *dnaE2* provides a modest increase in resistance to ciprofloxacin in wild-type biofilms compared with biofilms of the *dnaE2* knockout strain.

## Results

### Levels of ROS increased during *M. smegmatis* biofilm formation

**Figure 1:**
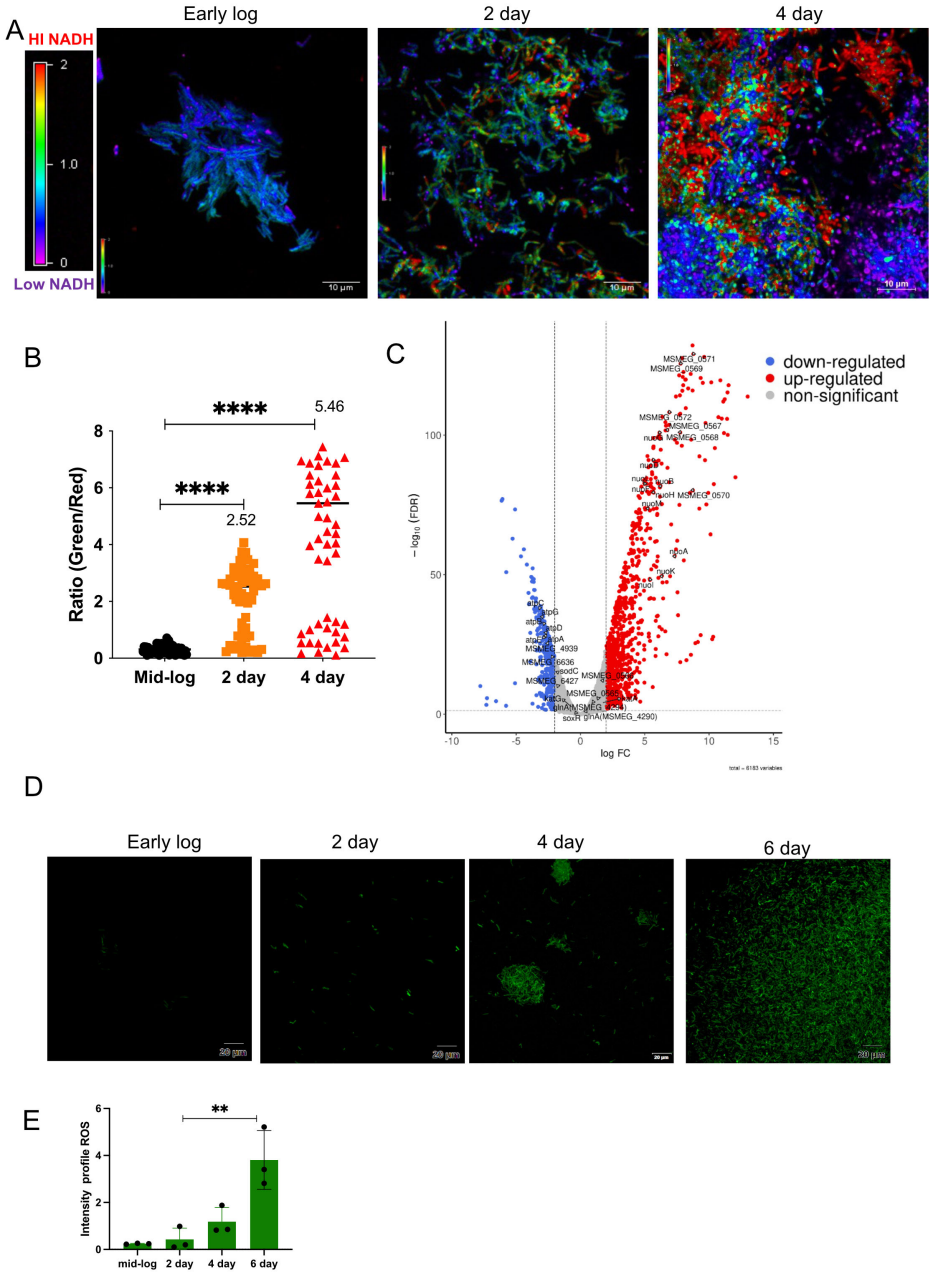
Determination of ROS levels in biofilm using a ROS indicator. **(A)** The intracellular redox status within the biofilm culture was determined using a Peredox–NADH biosensor. *M. smegmatis* mc^2^155 was seeded for biofilm formation with a sterile glass coverslip in 24-well plates, and NADH levels were monitored at 2 and 4 days, with early-log-phase culture as the control. Biofilm cultures were observed under a microscope. The images were assigned pseudo-colors to represent NADH/NAD^+^ levels in individual cells using a color heat map. The experiment was repeated at least twice, with two replicates each. **(B)** Quantification of NADH/NAD^+^ ratios from three independent fields containing 60 planktonic and 60 biofilm cells. Median values are plotted for 2 and 4 days. Variation between samples was analyzed using one-way ANOVA (****P < 0.0001). **(C)** Volcano plot depicting upregulated and downregulated genes on the 6^th^ day of biofilm culture compared with planktonic culture, analyzed by RNA-Seq. **(D)***M. smegmatis* mc^2^155 was seeded for biofilm formation in triplicate. After 2 and 4 days of incubation, cells were harvested, washed, stained with the CM-CH_2_DCFDA ROS indicator, and subjected to CLSM. Fluorescent images were acquired, and a representative image is shown. **(E)** Mean fluorescence intensity from three independent fields for each time point was plotted, and data were compared using one-way ANOVA (**P < 0.01).

There is a close connection between biofilm formation and the production of reactive oxygen species (ROS). Imbalances in NADH/NAD^+^ levels in cells often result in ROS production. To determine whether ROS was produced during mycobacterial biofilm formation, we monitored the NADH/NAD^+^ levels in the bacterial population using a *Mycobacterium*-optimized fluorescent NADH biosensor (Bhat et al., 2016). Microscopic analysis of the biofilm culture harboring the biosensor revealed a gradual increase in NADH levels during biofilm formation (**Figure 1A**, panels for the 2^nd^ and 4^th^ days; **Figure 1B**, quantitation of relative intensities). We also observed a high degree of variation in the NADH level of the 4^th^-day biofilm, which could arise as a consequence of local heterogeneity in the microenvironment and nutrient availability. Next, we compared the transcriptome of *M. smegmatis* during planktonic and 6^th^-day biofilm stages by performing RNA-Seq analysis on the isolated total RNA. We observed that the operon encoding NADH oxidation (*nuoA–nuoM*) was upregulated, whereas the gene cluster involved in ATP synthesis (*atpA–atpG*) was downregulated (**Figure 1C**, **Tables 1**, **2**; **Supplementary Data Sheet 1**). The *nuoA–nuoM* operon constitutes respiratory Complex I, through which the accumulating NADH in the biofilm is recycled. We reasoned that NADH recycling in the absence of ATP synthesis would be accompanied by the transfer of electrons to molecular oxygen, resulting in the generation of superoxide and, ultimately, an increase in ROS levels (Murphy, 2009; Xie et al., 2020). Additionally, the expression of genes involved in ROS detoxification, such as *katG*, *sodC*, and Mn-dependent superoxide dismutases (*MSMEG_6427* and *MSMEG_6636*), was downregulated in the biofilm culture (**Figure 1C**, **Table 2**; **Supplementary Data Sheet 1**). To validate this observation, we disrupted the biofilm culture and stained the bacteria with the ROS indicator CM-CH_2_DCFDA at different stages of biofilm maturation. We observed a gradual accumulation of ROS as the biofilm matured (**Figure 1D**, panels for the 2nd, 4^th^, and 6^th^ days; **Figure 1E**), consistent with the predicted outcome from the RNA-Seq and NADH biosensor data.

**Table 1:** Differential expression profile of NADH oxidase genes change, <0.5-down-regulated, >1.5 up-regulated).

Locus tag	Gene function	Gene name	Planktonic to biofilm	Biofilm to recovery
*MSMEG_2063*	NADH-quinone oxidoreductase, a subunit	*nuoA*	37.90	0.032
*MSMEG_2051*	NADH-quinone oxidoreductase, M subunit	*nuoM*	31.38	0.017
*MSMEG_2052*	NADH-quinone oxidoreductase, L subunit	*nuoL*	80.51	0.0065
*MSMEG_2053*	NADH-quinone oxidoreductase, k subunit	*nuoK*	42.32	0.0037
*MSMEG_2055*	NADH-quinone oxidoreductase, I subunit	*nuoI*	51.61	0.02
*MSMEG_2056*	NADH-quinone oxidoreductase, H subunit	*nuoH*	70.87	0.015
*MSMEG_2057*	NADH-quinone oxidoreductase, G subunit	*nuoG*	32.01	0.021
*MSMEG_2058*	NADH-quinone oxidoreductase, F subunit	*nuoF*	50.64	0.017
*MSMEG_2060*	NADH-quinone oxidoreducatase, D subunit	*nuoD*	74.89	0.00566
*MSMEG_2062*	NADH-quinone oxidoreductase, B subunit	*nuoB*	0.63	0.69

**Table 2:** Differential expression profile of ATP synthesis and ROS regulating genes Expression values (1-no change, <0.5-down-regulated, >1.5 up-regulated).

Locus tag	Gene function		Planktonic to biofilm	biofilm to recovery
*MSMEG_4936*	ATP synthase F1, beta subunit	*atpD*	0.16	16.56
*MSMEG_4938*	ATP synthase F1, alpha subunit	*atpA*	0.15	21.6
*MSMEG_4942*	ATP synthase F0, A subunit	*atpB*	0.14	15.4
*MSMEG_4937*	ATP synthase F1, gamma subunit	*atpG*	0.13	23.2
*MSMEG_4935*	ATP synthase F1, epsilon subunit	*atpC*	0.11	20.5
*MSMEG_6427*	[Mn] superoxide dismutase		0.29	1.084
*MSMEG_6636*	[Mn] superoxide dismutase		0.21	1.71
*MSMEG_4320*	alkyl hydroperoxide reductase/Thiol specific antioxidant/Mal allergen		0.97	1.68
*MSMEG_6384*	catalase/peroxidase HPI	*katG*	0.62	0.982
*MSMEG_0835*	copper/zinc superoxide dismutase	*sodC*	0.29	4.85

### The regulation of DNA repair and mutasome pathways was found to be divergent during the biofilm formation and subsequent recovery stages

Increased ROS levels result in DNA damage and induce DNA repair genes that are predominantly involved in the base excision repair pathway (Imlay, 2013). Therefore, we compared the expression profiles of DNA repair genes in total RNA from mature biofilms and recovery stages using RNA-Seq analysis. Surprisingly, genes involved in the GO repair pathway, such as *mutY and mutM*, and other DNA repair genes, such as *ung*, *nei, xth*, and *mfd*, were downregulated by 2–4-fold in mature biofilms. Interestingly, the error-prone polymerase *dnaE2* showed a reverse correlation; that is, the gene was induced during biofilm formation and downregulated during the recovery phase. Along with *dnaE2*, its accessory factors *imuA’* and *imuB*, which constitute the mutasome cassette in *M. smegmatis* and *M. tuberculosis*, were upregulated in the biofilm by 2–4-fold (**Tables 3**, **4**, planktonic to biofilm column).

**Table 3:** Genes involved in DNA replication that are differentially regulated during biofilm formation and recovery stages (1-no change, <0.5-down-regulated, >1.5 up-regulated).

Gene name	Common name	Gene function	Planktonic to biofilm	Biofilm to recovery
*MSMEG_6896*	*ssb*	single-stranded DNA-binding protein	0.08	23.34
*MSMEG_6285*	*dnaZX*	DNA polymerase III gamma/tau subunit	0.28	7.19
*MSMEG_6157*	*topA*	DNA topoisomerase I	0.35	3.13
*MSMEG_0456*		DNA gyrase subunit A	0.37	4.30
*MSMEG_0001*	*dnaN*	DNA polymerase III, beta subunit	0.36	3.05
*MSMEG_0457*		DNA topoisomerase IV subunit B	0.54	3.05
*MSMEG_3839*	*polA*	DNA polymerase I	0.52	2.62
*MSMEG_0006*	*gyrA*	DNA gyrase, A subunit	0.71	2.46
*MSMEG_6079*	*radA*	DNA repair protein RadA	0.50	2.83
*MSMEG_3885*	*helY*	DEAD/DEAH box helicase	0.31	7.25
*MSMEG_4572*		DNA polymerase III, delta subunit	0.56	2.92
*MSMEG_6153*		DNA polymerase III subunit delta	0.44	2.64
*MSMEG_6892*	*dnaB*	replicative DNA helicase	0.80	2.40
*MSMEG_2403*	*recG*	ATP-dependent DNA helicase RecG	0.45	2.30
*MSMEG_6443*		Hypothetical protein	6.20	0.42

**Table 4:** Genes involved in DNA repair that are differentially regulated during biofilm formation and recovery stages.

Gene name	Common name	Gene function	Planktonic to biofilm	Biofilm to recovery
*MSMEG_2399*	*ung*	uracil-DNA glycosylase	0.61	1.90
*MSMEG_2419*	*mutM*	formamidopyrimidine-DNA glycosylase	0.77	1.96
*MSMEG_4683*	*nei1*	putative formamidopyrimidine-DNA glycosylase	0.30	7.62
*MSMEG_6187*	*nth*	endonuclease III	0.60	2.42
*MSMEG_0829*	*xth*	exodeoxyribonuclease III	0.33	3.96
*MSMEG_2765*	*dut*	dUTP hydrolase	0.49	3.96
*MSMEG_5148*	*mutT2*	CTP pyrophosphohydrolase	0.26	2.55
*MSMEG_0790*	*mutT3*	hydrolase, NUDIX family protein	0.56	1.62
*MSMEG_6927*	*mutT4*	MutT/nudix family protein	0.53	2.21
*MSMEG_3078*	*uvrC*	excinuclease ABC, C subunit	0.68	2.40
*MSMEG_5423*	*mfd*	transcription-repair coupling factor	0.25	11.04
*MSMEG_3172*	*dinX*(*dinB1*)	DNA polymerase IV 1	0.58	4.64
*MSMEG_1633*	*dnaE2*	DNA polymerase III, alpha subunit, putative	2.12	0.75
*MSMEG_1620*	*imuA’’*		5.03	0.29
*MSMEG_1622*	*imuB*	putative DNA repair polymerase	2.74	0.57

Expression values (1-no change, <0.5-down-regulated, >1.5 up-regulated).

**Figure 2:**
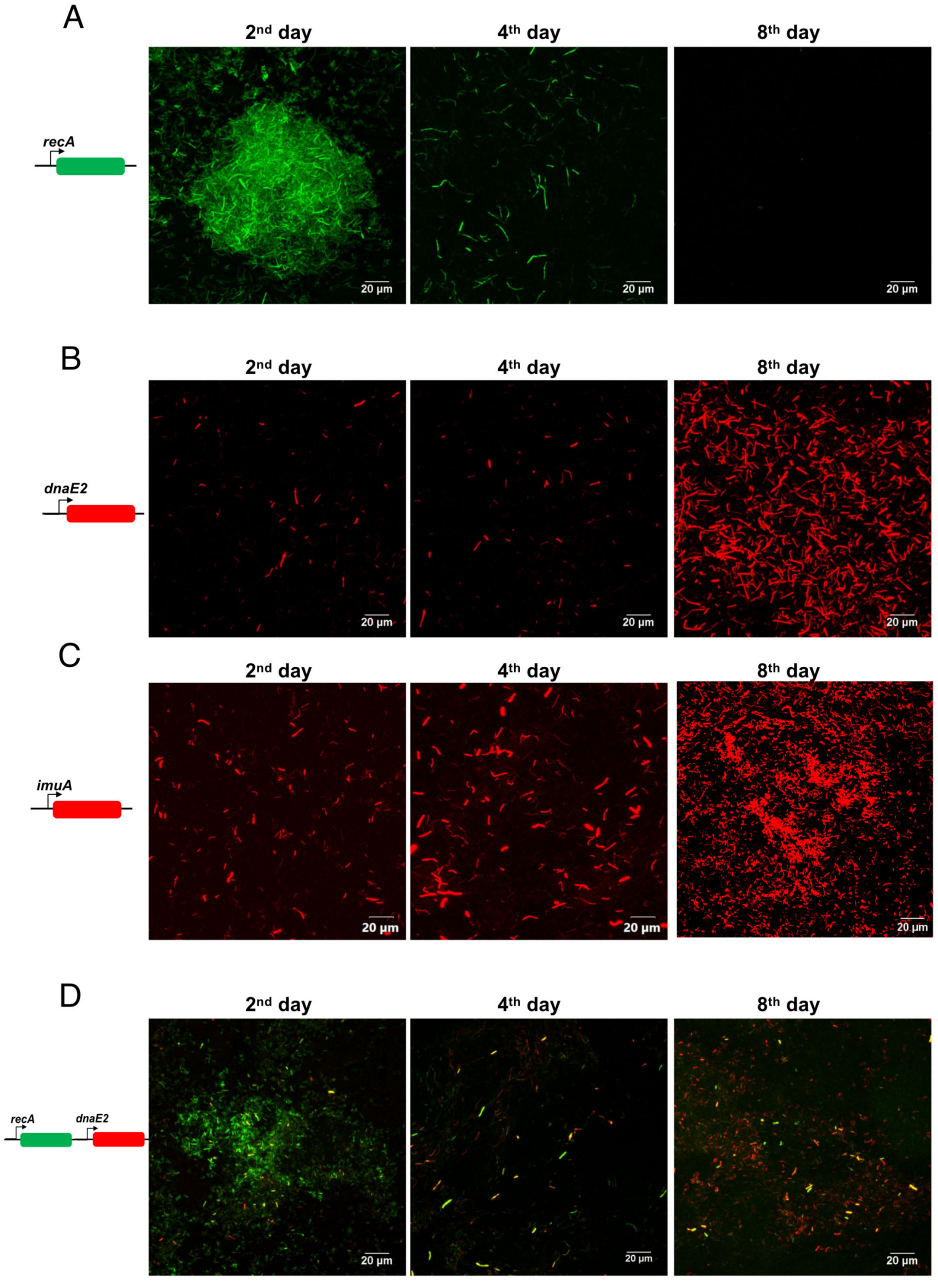
Expression analysis of *recA, dnaE2*, and *ImuA’* in the biofilm. **(A)***M. smegmatis* harboring pMV262 *P_recA_~*mClover was seeded for biofilm formation with a sterile glass coverslip in a 24-well plate and incubated for 8 days. After incubation, the medium was removed, and the biofilm layer on the coverslip was imaged. Fluorescent images were acquired at defined time points, and a representative image from triplicate experiments is shown. **(B)***M. smegmatis* with pMV262 *P_dnaE2_~*mCherry was seeded for biofilm formation as described above, and fluorescent images were acquired at defined time points. Representative images from triplicates are shown. **(C)***M. smegmatis* with pMV262 *P_ImuA’_~*mCherry was seeded for biofilm formation for 8 days, and fluorescent images were acquired at defined time points. A representative image from triplicates is shown. **(D)***M. smegmatis* harboring the dual reporter pMV262 *P_recA_~*mClover and *P_dnaE2_~*mCherry was seeded for biofilm formation and incubated for 8 days. Fluorescent images corresponding to the expression of *P_recA_* (green) and *P_dnaE2_* (red) were acquired at defined time intervals, and merged images are presented.

Using fluorescent reporter strains, we further examined the temporal expression of the mutasome and its upstream regulator *recA* during the different stages of biofilm formation. We generated a destabilized mClover reporter fused to the *M*. *smegmatis recA* promoter sequence (*P_recA_*). The *M*. *smegmatis* strain harboring the *P_recA_*~mClover reporter plasmid was seeded for biofilm formation, and reporter signals were collected every 2 days. We observed high recA expression in most cells of the biofilm (**Figure 2A**, panel for the 2^nd^ day), which progressively decreased as the biofilm matured (**Figure 2A**, panels for the 4^th^ and 8^th^ days; **Supplementary Figure S2A**, quantitation).

The expression of DnaE2, along with its accessory factors ImuA’ (MSMEG_1620) and ImuB (MSMEG_1622), results in mutagenesis during the SOS response in *M. smegmatis* and *M. tuberculosis* (Boshoff et al., 2003; Warner et al., 2010). Therefore, we examined the expression of *dnaE2* and *imuA’* at different stages of biofilm formation (**Figures 2B, C**, respectively) using gene-specific promoters fused to a destabilized version of mCherry. Microscopic analysis of the biofilm revealed that *dnaE2* expression was detected in fewer cells on the 2^nd^ day, which subsequently increased in the population as the biofilm matured (**Figure 2B**, panels for the 4^th^ and 8^th^ days; **Supplementary Figure S2B**, quantitation). The expression pattern of *imuA’* was considerably more widespread in the biofilm on the 2^nd^ day than that of *dnaE2*. However, the number of mCherry-positive bacteria increased after the 4^th^ day, similar to *dnaE2* (**Figure 2C**; **Supplementary Figure S2C**, quantitation). Because *imuA’* and *imuB* constitute an operon, the expression pattern of *imuB* could not be determined by reporter analysis. We speculate that the expression of *imuB* is similar to *that of imuA’*. Reporter expression revealed a delay between the expression of *recA* and its downstream target *dnaE2* (2^nd^ day for *recA* and 4^th^ day for *dnaE2* in **Figures 2A, B**, respectively). To validate that the observed temporal difference in the expression of *recA* and *dnaE2* was not due to experimental variation between the two strains, we generated a dual reporter strain harboring *P_recA_*~mClover and *P_dnaE2_*~mCherry and observed the expression patterns of the individual genes. In agreement with our reporter findings, the expression of *P_recA_*~mClover preceded that of *P_dnaE2_*~mCherry in the biofilm population (**Figure 2D**, panels for the 2^nd^ to 8^th^ days; **Supplementary Figure S2D**, quantitation). Deletion of *recA* resulted in a significant reduction in *dnaE2* expression in biofilms, in agreement with earlier reports (Boshoff et al., 2003) (**Supplementary Figure S1**, *recA::kan* panel). Because there is an alternate route for *dnaE2* induction through the PafBC pathway, we tested the expression of *P_dnaE2_* in the biofilm of *the PafBC* mutant. Although the expression was not comparable to that of the wild-type strain, the *pafBC* mutant strain showed widespread *dnaE2* expression in the biofilm compared with that in the *recA* knockout strain (**Supplementary Figure S1**, wild-type panel, and middle panel Δ*pafABC*). These findings demonstrate that *dnaE2* expression in biofilms occurs predominantly through the *recA* route and not through *pafBC*.

### DnaE2 expression contributed to bacterial heterogeneity in biofilm

**Figure 3:**
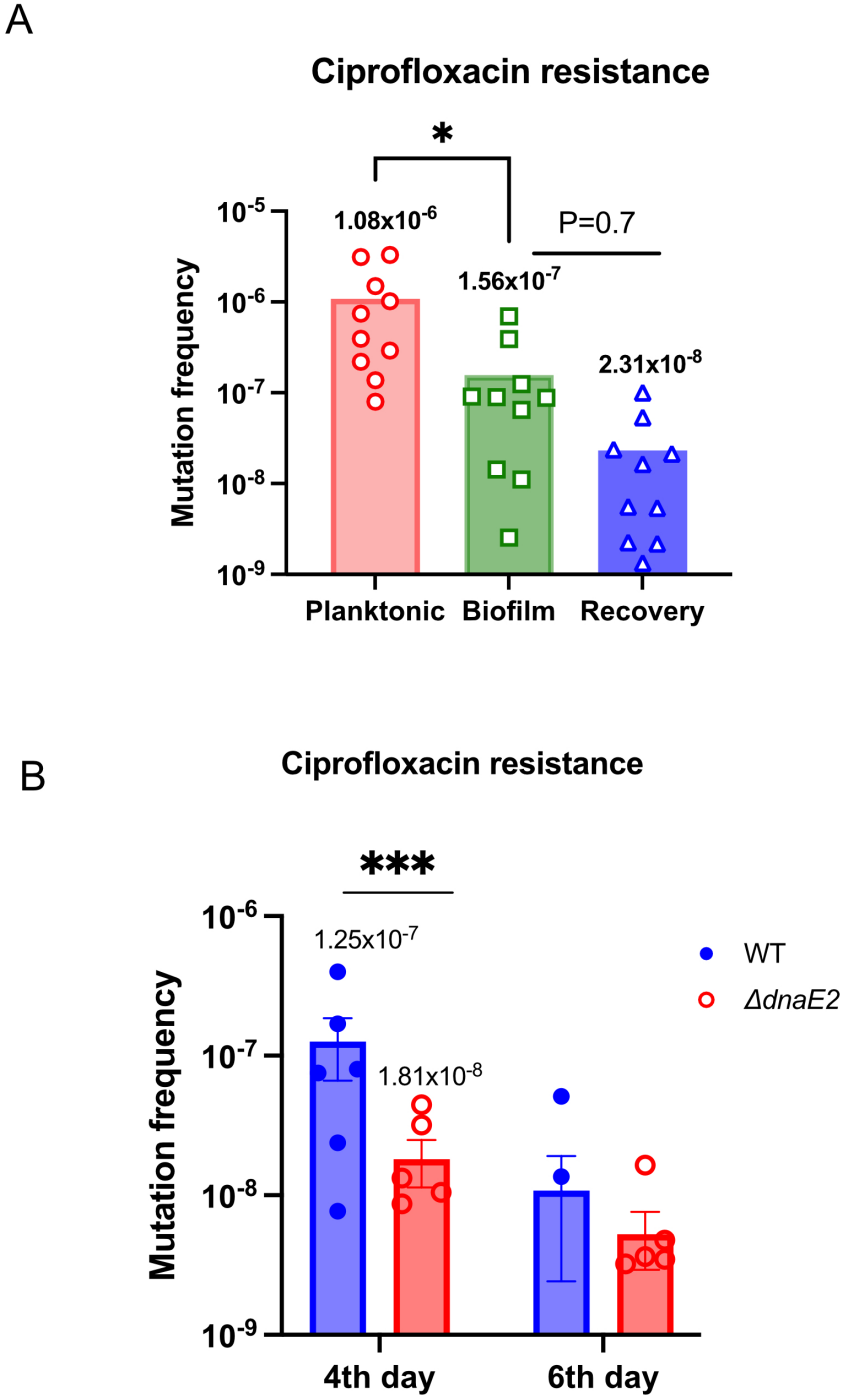
Contribution of *dnaE2* expression to bacterial heterogeneity in biofilms. **(A)** Ten replicates of 2 mL cultures of the WT strain from planktonic, 6^th^-day biofilm, and 6^th^-day biofilm that was mechanically disrupted and recovered in 7H9 medium for 3 h (biofilm-to-planktonic recovery stage) were harvested and plated on 7H10 plates containing ciprofloxacin to score for mutants. Small aliquots were plated to determine viable counts. Mutation frequency for each condition was determined, and individual values were plotted using GraphPad Prism^®^ v8. Differences between mean mutation frequencies were tested using Student’s *t*-test (P < 0.05). **(B)** Twelve biofilm cultures each of the WT and *ΔdnaE2* strains were harvested and plated on 7H10 plates containing ciprofloxacin to isolate mutants on the 4th and 6^th^ days. Small aliquots were serially diluted and plated on 7H10 plates without antibiotics for viable counts. Mutation frequency was calculated as the ratio of the number of mutants to the viable count for each replicate. Individual values were plotted on a scatter plot using GraphPad Prism^®^ v8, and the mean value for each set is indicated above the plot. For the 6^th^-day time point, only two of six WT replicates produced resistant colonies. Variances between 4^th^-day samples were tested using the F-test (*P* = 0.0002), P <0.5=*, ***P<0.001.

Transcriptome analysis of biofilms revealed that the expression of DNA repair enzymes was downregulated. Simultaneously, there was an increase in the expression of the mutasome consisting of *dnaE2*, *imuA’*, and *imuB* (**Table 4**, **Figure 2**). This observation led to the hypothesis that these two factors can synergistically promote mutagenesis in biofilm cultures. We determined the mutation frequency between planktonic, biofilm, and recovery stages in the wild-type (WT) strain by scoring the occurrence of spontaneous resistant colonies on ciprofloxacin plates (**Figure 3A**). Interestingly, the mean mutation frequency of the planktonic stage was significantly higher (1.08 × 10^–6^) than that of the biofilm stage (1.56 × 10^-7^). The mean mutation frequency in the recovery stage was reduced by nearly sevenfold compared with that in the biofilm stage [**Figure 3A**; from 1.56 × 10^-7^ in biofilm (green bar) to 2.31 × 10^-8^ during recovery (blue bar)]. We next compared the mutation frequencies of the WT and *ΔdnaE2* strains at different stages of biofilm maturation (4^th^ and 6^th^ days). As shown in **Figure 3B**, the average mutation frequency of the WT biofilm was nearly sevenfold higher than that of *ΔdnaE2* [WT (1.25 × 10^–7^) vs. *ΔdnaE2* strain (1.8 × 10^–8^)] on the 4th day, while on the 6th day, the difference in mutation frequency was reduced to approximately twofold [WT, 1.07 × 10^–8^) vs. *ΔdnaE2* strain (5.2 × 10^–9^)]. We believe that this apparent discrepancy could be due to the increased sensitivity of newly released (NRel) bacteria from biofilms to antibiotics, which has been reported in *the Haemophilus* and ESKAPE groups of bacteria, in addition to the presence of dead cells within the biofilm. While characterization of the NRel phenomenon is beyond the scope of this study, we performed live–dead staining using Syto9 and propidium iodide on the 6^th^ day of culture and observed a significant number of dead bacteria in the biofilm population (**Supplementary Figure S3A**, planktonic vs. biofilm panels). The percentage of live cells was nearly 93% for the planktonic culture and approximately 35% for the biofilm culture (**Supplementary Figure S3B**). We also observed a time-dependent increase in bacterial death, as measured by eDNA release, with the maximum level of cell death observed on the 6^th^ day (**Supplementary Figure S3C**). We believe that the number of dead bacteria, along with NRel, could contribute to the observed reduction in mutation frequency on the 6^th^ day. To overcome these limitations, we developed an alternate strategy to detect mutations in the biofilm using a GFP reversion assay, in which a non-fluorescent variant of GFP (GFP*) with a +1 frameshift in the open reading frame (ORF) (**Supplementary Figure S4A**) regains fluorescence through a compensatory mutation. Transformants of *M. smegmatis* mc^2–^155 and *ΔdnaE2* harboring GFP* were subjected to biofilm formation and observed for the appearance of green fluorescence. While the reversion rate of GFP* was very low even in the WT biofilm, fluorescence intensity was noticeably higher and more consistent than that of the *ΔdnaE2* strain (**Supplementary Figure S4B**, top vs. bottom panels).

**Figure 4:**
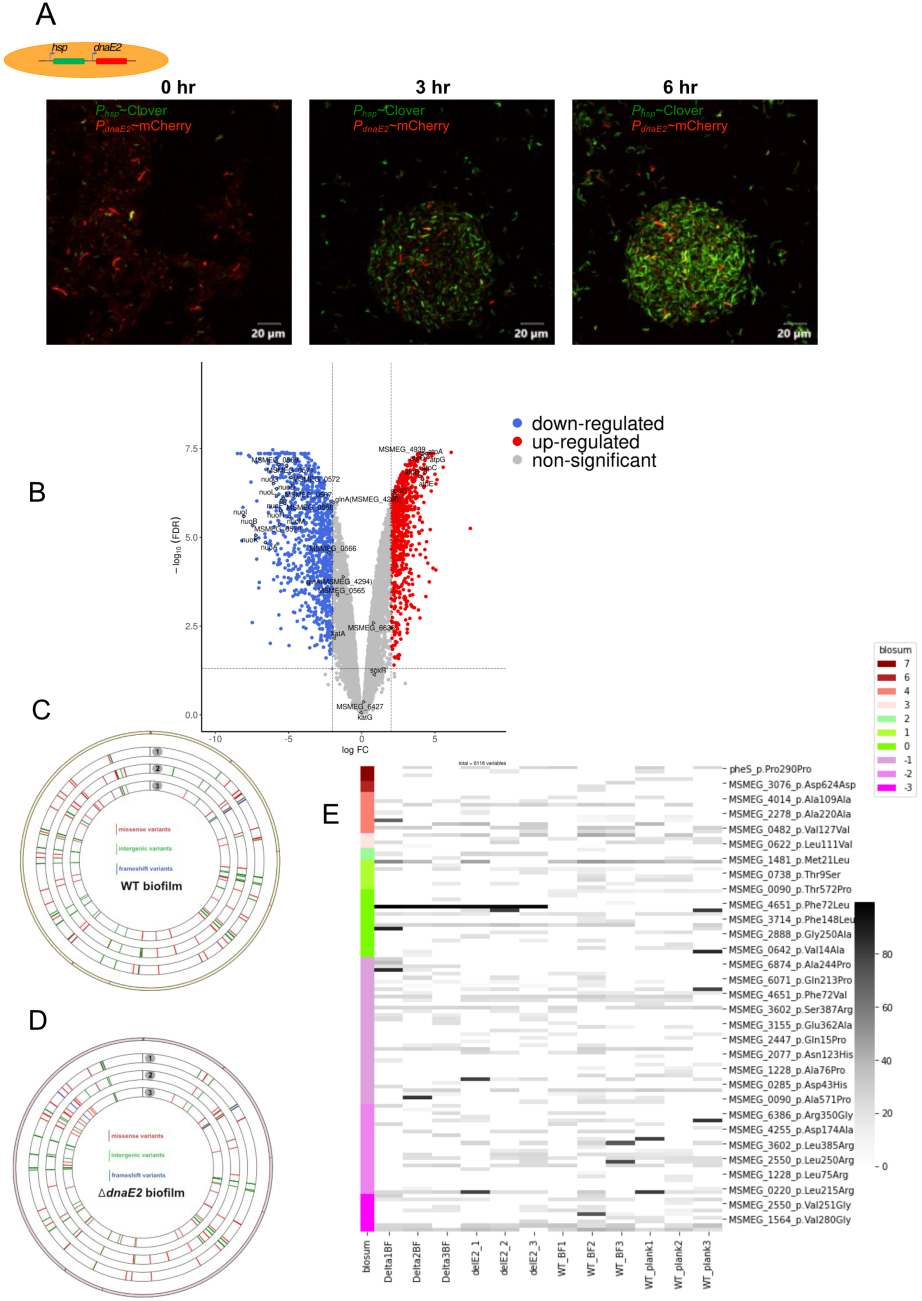
Gene expression during biofilm-to-planktonic transition and impact on genome-wide mutagenesis. **(A)** Time-course analysis of *dnaE2* expression during the biofilm recovery stage of *M. smegmatis* using *P_hsp_*~mClover and *P_dnaE2_*~mCherry dual reporter strains in 7H9T medium. **(B)** Volcano plot of upregulated and downregulated genes during the recovery stage compared with biofilm culture, analyzed by RNA-Seq. **(C)** Circos plot representing SNPs identified in WT biofilm cultures. The outermost circle represents the reference genome, with SNPs marked in the clockwise direction. Tracks 1, 2, and 3 represent biological triplicates of WT biofilm cultures. Red bars indicate missense/non-synonymous mutations, green bars indicate intergenic mutations, and blue bars indicate frameshift mutations. **(D)** Circos plot representing SNPs identified in *ΔdnaE2* biofilm cultures. The outermost circle represents the reference genome, with SNPs marked in the clockwise direction. Tracks 1, 2, and 3 represent biological triplicates of Δ*dnaE2* biofilm cultures. Red bars indicate missense/non-synonymous mutations, green bars indicate intergenic mutations, and blue bars indicate frameshift mutations. **(E)** Heat map representing SNPs identified in WT and *ΔdnaE2* strains under planktonic and biofilm culture conditions. A blossom score analysis represents amino acid changes. From left to right: columns 1–3, *ΔdnaE2* biofilm cultures; columns 4–6, *ΔdnaE2* planktonic cultures; columns 7–9, WT biofilm cultures; and columns 10–12, WT planktonic cultures. The intensity of each bar represents the percentage occurrence of mutated alleles.

Previously, we observed that the expression of *dnaE2* in antibiotic-treated *M*. *smegmatis* persister cells persisted for nearly 30h after recovery in antibiotic-free medium (Salini et al., 2022). Similarly, we monitored the dynamics of *dnaE2* expression during the biofilm-to-planktonic transition (recovery) by mechanically disrupting the biofilm and culturing it in 7H9 medium with Tween 80 to prevent bacterial aggregation. Time-course microscopic analysis of the biofilm-to-planktonic transition culture revealed that within 3h of the recovery phase, there was a significant reduction in mCherry-expressing cells (*P_dnaE2_*) and reduced further decreased by 6h (**Figure 4A**, panels ii and iii). RNA-Seq analysis confirmed this observation and revealed that DNA repair genes downregulated during biofilm formation were induced to near planktonic levels. Furthermore, expression of the *nuoA–nuoM* gene cluster was downregulated, whereas the *atpA–atpG* cluster was upregulated, representing a complete reversal of the biofilm state (**Figure 4B**, **Tables 1**, **2**; **Supplementary Data Sheet 2**).

We designed a long-term experiment in which three independent replicates of the wild-type (WT) and Δ*dnaE2* strains were passaged three times in biofilms, followed by whole-genome sequencing (WGS) analysis of the biofilm cultures. This analysis allowed us to identify single nucleotide polymorphism (SNP) changes occurring across the genome and compare them with those in planktonic cultures. As shown in the Circos plots (**Figures 4C–E**), SNPs were detected across the genomes of both the WT and *ΔdnaE2* strains.

Interestingly, the variation in the distribution of SNPs in genes involved in metabolic and other physiological functions between the planktonic cultures of WT and *ΔdnaE2* was insignificant (**Supplementary Figures S5A, B**). Furthermore, analysis of the genes within these regions revealed that the observed SNPs mapped to genes with important cellular functions, including metabolism, transporter proteins, transcriptional regulators, and essential proteins involved in *the esx-3* secretion system (*MSMEG_0622*) and cell division (*ftsQ*) (**Figure 4E**; **Supplementary Data Sheet 3**). We also observed SNP patterns in the coding regions of genes associated with various metabolic pathways. For example, SNPs in genes involved in alternative metabolism and sulfate metabolism (*MSMEG_1564*, *MSMEG_2888*, and *cysT*) were observed only in planktonic cells. Likewise, SNPs in the membrane transporter *mmpL11* were detected in both planktonic and biofilm cultures of the WT strain. In contrast, specific SNPs were observed only in *the ΔdnaE2* strain, irrespective of the culture condition, including MSMEG_2038 and *MSMEG_1693* (succinate dehydrogenase). Furthermore, SNPs in the shikimate transporter MSMEG_6332, the integral membrane transport protein MSMEG_6818, and MSMEG_0220, annotated as dehydrogenase and monoglyceride lipase, were found exclusively in the biofilm culture of *the ΔdnaE2* strain, suggesting that these mutations may play a role in the survival of the *ΔdnaE2* strain under biofilm conditions (**Supplementary Data Sheet 3**). Additionally, SNPs were identified in two-component systems and transcriptional regulators (*prrA*, *MSMEG_0166*—a GntR family transcriptional protein), glyoxylate reductase, lipase, and genes involved in cell wall synthesis and membrane transport systems, such as MmpL11 (MSMEG_0241) and *aftA* (arabinofuranosyltransferase) (**Supplementary Data Sheet 3**).

### *dnaE2* was required for bacterial fitness in biofilm culture

**Figure 5:**
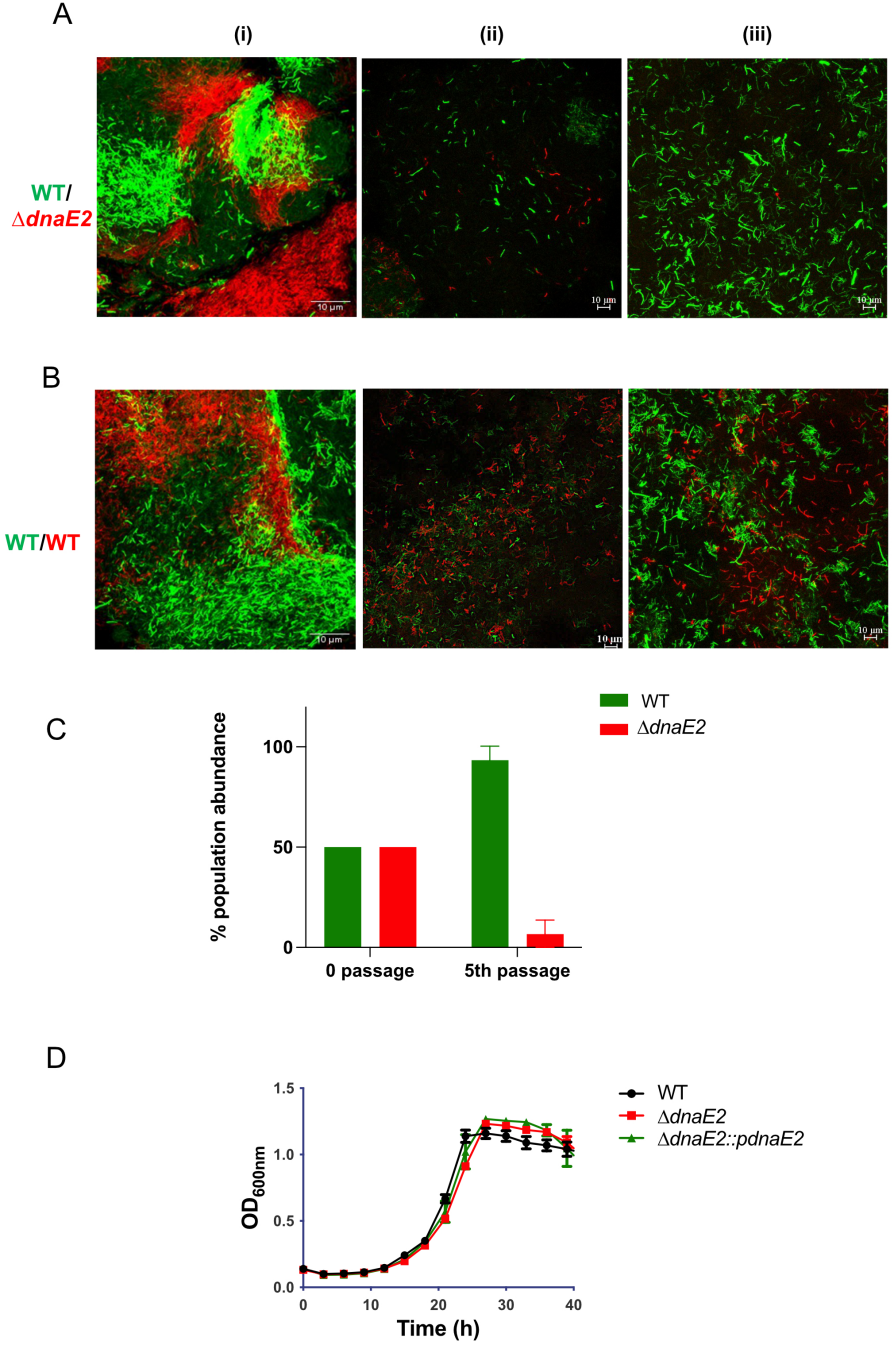
Competition between WT and *ΔdnaE2* biofilm populations. **(A, B)***M. smegmatis* WT and *ΔdnaE2* strains expressing short half-life variants of mClover and mCherry, respectively through *P_hsp_* were mixed in equal proportions and co-cultured continuously for five passages. Microscopic images of the mixed populations were obtained at the 1^st^ and 5^th^ passages. The experiment was repeated at least twice. **(C)** Population abundance of WT and *ΔdnaE2* strains at the beginning and 5^th^ passage, plotted from three replicates. Representative graphs from two independent experiments are shown. The experiment was repeated twice, with two replicates. **(D)** Growth profiles of *M. smegmatis* strains in 7H9T medium. Five replicates of each strain were diluted to a final OD_600_ of ~0.02 and seeded into individual wells of a Honeywell Comb plate. The plate was incubated at 37°C with constant shaking, and growth was monitored by measuring absorbance at 600 nm using a Bioscreen C instrument (Bioscreen, Tecan, Finland).

Next, we determined the fitness of the *dnaE2* mutant strain compared with that of the wild-type (WT) strain by co-culturing them within the biofilm. To perform this competition study, we generated fluorescently labeled strains of WT and *ΔdnaE2* encoding mClover and mCherry, respectively, under the control of the *P_hsp_* promoter. Cultures containing equal proportions of WT and *ΔdnaE2* were allowed to form biofilms, and population density was observed microscopically. During the initial stages, the bacterial population density was approximately 50% for both the WT and *ΔdnaE2* strains (**Figure 5A**, panel i; **Figure 5C**, 0 passage). However, upon repeated passaging, the density of *ΔdnaE2* (red bacteria) drastically diminished to approximately 5% by the fifth passage in the biofilm (**Figure 5A**, panels ii–iii; **Figure 5C**, 5^th^ passage). To rule out the potential impact of mClover or mCherry on bacterial fitness, we competed WT strains harboring mClover and mCherry and did not observe a significant reduction in population density at the 5^th^ passage (**Figure 5B**, panels i–iii). To determine whether the mutant strain exhibited any growth defects, we examined the growth kinetics of WT, *ΔdnaE2*, and complemented strains in liquid medium. There was no significant reduction in the growth rate of the *ΔdnaE2* strain when cultured independently in liquid medium (**Figure 5D**). These findings indicate that the observed fitness defect of the *ΔdnaE2* strain in biofilm co-culture with WT is not due to an inherent growth defect.

In a 21-day experiment, we observed that in the *ΔdnaE2* strain, the pellicle formed at the liquid–air interface became unstable and settled at the bottom, whereas it remained intact in the WT and complemented strains (**Supplementary Figure S6A**, 6^th^-day time point; **Supplementary Figure S6B**, 21^st^-day time point, red arrows).

## Discussion

**Figure 6:**
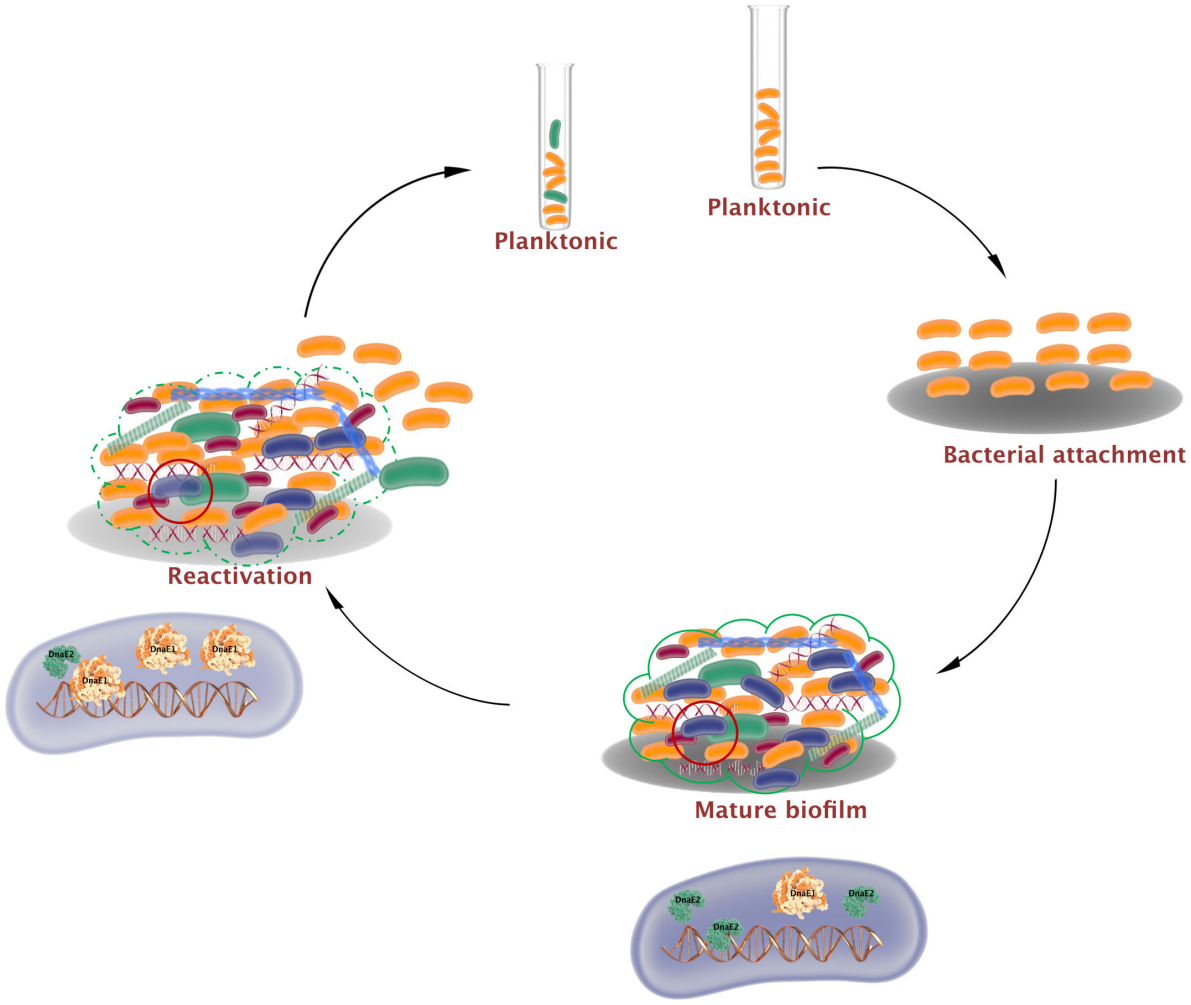
Mutagenesis and bacterial growth arrest by *dnaE2* expression in biofilms. Biofilm formation is initiated by the attachment of mycobacteria to a substratum. During maturation, an extracellular matrix encloses a heterogeneous population consisting of dead bacteria (red), growth-arrested bacteria (blue), and mutant bacteria (green) generated by reduced DNA repair and induction of the error-prone polymerase DnaE2. Growth arrest within this subpopulation arises from conflict between DnaE2 and the housekeeping polymerase DnaE1. During reactivation from biofilm to planktonic growth, DnaE2 expression is repressed, shifting the balance toward DnaE1 and resulting in active multiplication.

The intricate three-dimensional architecture of biofilms generates microenvironments that cause bacteria to display both chemical and genetic heterogeneity (Stewart and Franklin, 2008; Kolter et al., 2015; Sadiq et al., 2017). In the present study, we investigated the involvement of mycobacterial error-prone polymerases in generating genetic mutations in biofilms. Our findings show that during biofilm formation, ROS levels increase, inducing the SOS response and resulting in the expression of DnaE2. Reduced DNA repair and increased DnaE2 expression can synergistically promote mutagenesis in biofilms. Additionally, DnaE2 binding to genomic DNA may interfere with the function of the replication polymerase DnaE1, leading to reduced bacterial growth. However, when biofilms are mechanically disrupted and subjected to planktonic growth, DNA repair enzymes and replication-associated proteins are induced. Furthermore, DnaE2 expression during this transition phase is reduced, promoting bacterial growth (**Figure 6**).

Many bacteria form biofilms in response to stressors such as antibiotics and oxidative agents, which induce the SOS response (Gotoh et al., 2010; Oh et al., 2016; Cepas et al., 2019). To further understand the role of the SOS response and its contribution to mutagenesis, RNA-Seq analysis was performed on biofilm and recovered cultures of *M. smegmatis*. We observed induction of *recA* and the mutasome during biofilm formation (**Figure 2**, **Table 4**). We were intrigued by the induction of SOS regulon genes in the biofilm, as no additional stress was applied to the culture. Gene expression analysis showed increased expression of NADH oxidase enzymes, accompanied by a decrease in ATP synthesis, resulting in elevated ROS levels during biofilm formation (**Figures 1B, C**). ROS production has also been observed in yeast and other bacterial biofilms. In *Saccharomyces*, ROS production results from a metabolic shift from acidic to alkaline pH (Čáp et al., 2012). In *Candida*, farnesol—a signaling molecule involved in quorum sensing—induces ROS production, and in *Saccharomyces cerevisiae*, ROS arises through inhibition of mitochondrial electron transport (Machida et al., 1998; Čáp et al., 2012). In *Streptococcus pneumoniae* biofilms, the expression of *spxB* increases hydrogen peroxide production through pyruvate oxidation (Regev-Yochay et al., 2007). A similar mechanism has been reported in the biofilms of *Streptococcus sanguinis* and *Streptococcus gordonii* (Kreth et al., 2009). DNA damage, including strand breaks and base modifications resulting from hydrogen peroxide and other ROS in *Pseudomonas* and other bacterial biofilms, has been implicated in genetic variation (Boles et al., 2004). ROS has also been proposed to function as a signaling molecule that induces biofilm formation (Čáp et al., 2012; Gambino and Cappitelli, 2016). In *Mycobacterium avium*, the presence of autoinducer molecules can increase ROS levels and stimulate biofilm formation (Geier et al., 2008). Moreover, ROS can contribute to the stability of *Streptococcus* spp. biofilms by stimulating the release of eDNA, a major component of the biofilm matrix (Kreth et al., 2009; Aldecoa et al., 2017). Yang et al. reported that expression of the Gln-dependent regulon (*MSMEG_0565–0572*) during biofilm formation confers resistance to peroxide and aids nitrogen assimilation (Yang et al., 2018). Consistent with their findings, our RNA-Seq analysis revealed that the same genes were highly upregulated during biofilm formation (**Figure 1C**).

The potential role of ROS in the release of eDNA in *Mycobacterium* species has not yet been explored; however, it is known to cause DNA damage, including strand breaks and base oxidation.

Mycobacterial genomes, being GC rich, are highly sensitive to guanine oxidation, resulting in the formation of 8-oxoguanine residues that can interfere with replication. To overcome DNA damage, the induction of DNA repair mechanisms would be expected during biofilm formation. However, DNA repair enzymes belonging to the base excision repair pathway were downregulated, whereas the error-prone polymerase *dnaE2* and its accessory factors were upregulated (**Table 4**, biofilm). Interestingly, the expression of *recA*, the master regulator of the SOS response necessary for *dnaE2* induction, was significantly downregulated on the 4^th^ day and subsequent time points (**Figures 2A, D**, **Table 4**). This observation agrees with the findings of Yang et al., who showed that *recA* expression increased by approximately eightfold during the early stages of biofilm development but decreased to about 0.7-fold at later stages (Yang et al., 2017). Furthermore, *dnaE2* expression became widespread in the biofilm population from the 4^th^ to the 8^th^ day, showing an inverse relationship with recA expression. To understand the rationale behind this inconsistency, we searched for potential regulators that could induce *dnaE2*. The transcriptional regulator *pafBC* has been identified as a *RecA-independent* activator of *dnaE2* expression. However, reporter gene expression analysis of *dnaE2* in the *pafABC* knockout revealed that expression was not affected (**Supplementary Figure S1**, middle panel). Members of the *Mycobacterium* genus also encode additional translesion polymerases, such as DinB1 and DinB2, which can induce mutations through homopolymeric extension and frameshifts (Dupuy et al., 2023). In our study, the expression of *dinB1* and *dinB2* was either repressed or remained unchanged (**Table 4**; **Supplementary Data Sheet 1**), indicating that the contribution of these mutagenic polymerases was minimal.

Oxidative damage within biofilms has been shown to cause increased mutagenesis in *Pseudomonas* and *Staphylococcus* biofilm communities (Boles and Singh, 2008; Ryder et al., 2012). Furthermore, mutations within biofilms can result in the *de novo* emergence of drug-resistant and stress-tolerant variants that gain a competitive advantage (Bernier et al., 2013; Penterman et al., 2014; Steenackers et al., 2016). Because mutagenesis in mycobacterial biofilms has been relatively underexplored, we determined the mutation frequency by scoring spontaneous resistant mutants to Cip^R^ in biofilm cultures using a time-course experiment. We observed that at the earlier time point (4^th^-day biofilm), the difference in mutation frequency between the wild-type (WT) and *dnaE2* was considerable (approximately sevenfold). However, this difference became insignificant by the 6^th^ day (**Figure 3B**). Considering the synergistic effect of reduced DNA repair and increased mutasome expression in biofilms, the reduced mutation frequency on the 6^th^ day was unexpected (Conibear et al., 2009; García-Castillo et al., 2011). Several factors could contribute to this phenomenon: (i) the complex biofilm architecture and spatiotemporal variations in mutasome gene expression during biofilm formation, and (ii) increased cell death in the bacterial population, that would also contain the Cip^R^ mutants, which would result in an apparent reduction in mutation frequency. The results of live/dead staining of the biofilm support this interpretation (**Supplementary Figure S3**). (ii) In antibiotic-based mutation assays, only mutations that confer resistance yield colonies; however, ciprofloxacin resistance often carries a high fitness cost (Brandis et al., 2015; Melnyk et al., 2015) and such mutants may be outcompeted during biofilm development. Recent studies have shown that bacteria released from biofilms (NRel forms) are physiologically distinct and more sensitive to antibiotics than their planktonic counterparts (Mokrzan et al., 2020; Kurbatfinski et al., 2023). In our study, mutation frequency was determined by counting antibiotic-resistant colonies. We hypothesized that *M. smegmatis* released from biofilms by mechanical disruption would behave similarly to “newly released” cells, displaying higher antibiotic susceptibility, which could complicate the interpretation of resistance-based assays. Although the characterization of these released bacteria is beyond the scope of this study, if confirmed, our work would represent the first report of NRel forms in *the Mycobacterium* genus.

Another important observation from this study is the rapid restoration of DNA repair processes accompanied by a reduction in mutation burden when the biofilm was mechanically disrupted and cultured in planktonic medium (**Figure 3A**, **Table 4**). This “biofilm-to-planktonic transition” may reflect the state of DNA repair in bacteria dispersing from biofilms to repopulate as a planktonic founder population. While the increased antibiotic sensitivity of this “transition phase” due to the NRel phenotype may also contribute to the reduced mutation frequency, a sevenfold reduction in the mean mutation frequency during the recovery phase compared with biofilm culture (**Figure 3A**; reduction from 1.56 × 10–^7^ to 2.31 × 10^–8^) provides strong evidence of enhanced DNA repair during recovery. During natural biofilm dissolution and dispersion, it would be advantageous for such founder populations to possess robust DNA repair mechanisms to maintain genomic stability rather than a high mutation rate.

To understand the significance of *dnaE2* expression in biofilm cultures, we competed the mutant strain with the parental strain by co-culturing them in biofilms. While the mutant did not display significant growth defects when cultured individually in the planktonic state, there was a drastic reduction in the population density of the mutant compared with the parental strain after the 5^th^ passage in the biofilm (**Figure 5A**, panels ii–iii; **Figure 5C**). In a genome-wide transposon mutant library screening of *Mycobacterium tuberculosis* mutants showing biofilm formation defects, loss of *dnaE2* had only a minor impact on pellicle formation compared with the *relA* mutant, which had a much more severe defect (Richards et al., 2019). While the *relA* mutant is known to affect glycopeptidolipid (GPL) synthesis required for anchorage (Gupta et al., 2015), the role of *dnaE2* in biofilm development remains unclear. We reasoned that *dnaE2* expression could introduce beneficial mutations, resulting in higher fitness of the wild-type (WT) strain compared with the *ΔdnaE2* strain. A similar competition experiment involving the complemented strain would have been most desirable to demonstrate the role of *dnaE2* in conferring a fitness advantage. However, this experiment could not be performed due to the lack of plasmids with suitable selectable markers and compatible origins of replication to co-exist with the pMV261 plasmid expressing the fluorescent protein, as well as cross-resistance conferred by apramycin resistance in the complement strain. Until an appropriate method is developed to overcome this technical limitation, this aspect of the competition assay remains a limitation of the present study. To determine the true impact of *dnaE2* on bacterial mutagenesis, we performed whole-genome sequencing (WGS) of genomic DNA from biofilm cultures and observed allelic variants in both WT and *ΔdnaE2* from planktonic and biofilm conditions (**Figures 4C–E**; **Supplementary Figures S5A, B**). WGS analysis of biofilm cultures revealed mutations in several important metabolic enzymes and proteins implicated in bacterial survival (**Figure 4E**; **Supplementary Data Sheet 3**). This long-term experiment was not repeated to determine the reproducibility of the single nucleotide polymorphisms (SNPs). Given the spontaneous nature of mutations, it is unlikely that identical SNPs would appear upon repetition. Another limitation of this experiment is that the physiological effects of the identified SNPs could not be determined. We observed nucleotide changes in essential genes such as *ftsQ, aftA* (arabinofuranosyltransferase), *and esx-3*, as well as in sulfur and lipid metabolism enzymes. Although our data cannot confirm whether these mutations are beneficial or deleterious, a 5,000-passage long-term evolution experiment in *Salmonella* indicated a trend of reduced bacterial fitness, with more pronounced defects in strains lacking DNA repair enzymes involved in uracil excision and 8-oxoguanine excision pathways (Lind and Andersson, 2008). In our study, loss of *dnaE2* similarly resulted in reduced fitness when competing against the WT strain. We also observed that the pellicle in the *ΔdnaE2* strain became unstable and settled at the bottom in a 21-day biofilm stability experiment, whereas the WT strain remained stable at the liquid–air interface (**Supplementary Figures S6A, B**). The physiological implications of spontaneous pellicle disintegration are currently unknown, although one speculation is that pellicle collapse could trigger biofilm dispersion and transition to planktonic growth (**Figure 6**, last step of the biofilm-to-planktonic state). Reductions in second messenger molecules such as cyclic di-GMP or the production of small amounts of nitric oxide have been shown to represent “active modes” of biofilm dispersion in other bacteria. In contrast, “passive modes” of dispersion often result from abrasion, liquid shearing, or sloughing (Wille and Coenye, 2020) Currently, the mechanisms governing mycobacterial biofilm dissolution remain poorly understood. From our RNA-Seq data, we observed strong induction of resuscitation-promoting factor followed by genes involved in ribosome and ATP synthase synthesis, suggesting that bacteria enter an active growth phase after exiting the metabolically inactive or persister state.

Because the contribution of DnaE2 to mutagenesis was modest, we reasoned that additional roles of DnaE2 could be important for bacterial survival in biofilms. Previous studies have indicated that in *Escherichia coli*, expression of Pol IV impacts DNA synthesis (Indiani et al., 2009), and expression of DinB2 under a conditional promoter reduces *M. smegmatis* viability (Dupuy et al., 2023). Oxidative DNA damage results in 8-oxoguanine formation, which can stall the replisome and may require translesion synthesis activity by DnaE2. However, DnaE2 association with genomic DNA during translesion synthesis may create a “replication conflict” with the housekeeping polymerase DnaE1, generating a persister subpopulation characterized by slow growth. Although experimental evidence for this hypothesis is currently unavailable, induction of the SOS response has been reported to trigger persister formation in diverse bacterial groups (Podlesek and Bertok, 2020).

Until recently, the significance of mycobacterial biofilms was underappreciated because they were studied mainly *in vitro*. However, the detection of *M. tuberculosis* biofilms *in vivo* offers new insights into the pathogen’s ability to persist within the host (Chakraborty et al., 2021). Antibiotic therapy and the evolution of clinically relevant drug resistance in *M. tuberculosis* are often viewed as a “cause-and-effect” paradigm. Our study highlights that biofilms serve as a fertile niche for mycobacterial persistence and accelerate evolutionary processes in an antibiotic-independent manner driven by mutasome expression.

## Materials and methods

### Bacterial strains and culture conditions

The list of strains, plasmids, and oligonucleotides used in this study is presented in **Supplementary Tables 1**-**3**. Glycerol stocks of *Mycobacterium smegmatis* strains were revived on 7H10 Middlebrook agar supplemented with 0.5% (v/v) glycerol and 0.05% (v/v) Tween 80 containing appropriate antibiotics. Middlebrook 7H9 medium supplemented with 0.1% (v/v) glycerol and 0.05% Tween 80 (7H9T), along with appropriate antibiotics, was used for liquid cultures. When required, *M. smegmatis* cultures were selected using kanamycin (K; 50 µg/mL), apramycin (A; 25 µg/mL), or hygromycin (H; 50 µg/mL). *Escherichia coli* TG1 strains were used for cloning and plasmid DNA manipulation and were cultured in Luria–Bertani (LB) broth or LB agar at 37°C. For *E*. *coli* selection, the following antibiotic concentrations were used: kanamycin (50 µg/mL), apramycin (50 µg/mL), hygromycin (150 µg/mL), and ampicillin (100 µg/mL). All media components were procured from Difco (Maryland, USA), unless otherwise specified. Unless stated otherwise, all chemicals and reagents were obtained from Sigma-Aldrich (Missouri, USA).

### Determination of reactive oxygen species in mycobacterial biofilm

Freshly revived *M. smegmatis* mc^2–^155 wild-type (WT) cultures from 7H10 plates were seeded for biofilm formation in 7H9 biofilm medium. A bacterial suspension with an OD_600_ of 2.0 was prepared in biofilm medium and diluted 1:100 (in triplicate) into individual wells of a 24-well sterile plate (Nunc, Denmark) containing 2 mL of biofilm medium. The biofilm cultures were incubated at 30°C under static conditions. In parallel, a planktonic culture was prepared in 7H9T medium with an initial OD_600_ corresponding to 0.05–0.1 and incubated at 37°C in a shaker incubator at 175 rpm. Planktonic cultures in early log phase (OD_600_ ~0.3–0.4) were used as negative controls.

After 2 and 4 days of incubation, the biofilms were disrupted by vigorous pipetting. The cells adhering to the wells were collected by washing with 7H9 medium containing 0.2% Tween 80 (7H9–high Tween medium). The bacterial cells were harvested by centrifugation at 13,000 rpm for 1min, washed twice with 7H9–high Tween 80 medium, and resuspended in 90 µL of 1X PBS with 0.2% Tween 80 (PBS–high Tween).

The ROS indicator dye CM-H_2_DCFDA (Invitrogen, California, USA) was prepared by dissolving the dried powder in 100 µL of 100% DMSO to obtain an ~80 mM stock solution. This solution was added to the bacterial suspension in PBS–high Tween at a final concentration of 8 µM. The bacterial samples were incubated in the dark for 30 min at 37°C. After incubation, the samples and planktonic controls were concentrated by mild centrifugation, and the excess broth was discarded. The bacterial suspension with residual medium was mounted on glass slides with ProLong Glass Antifade Mountant (Thermo Fisher Scientific, Oregon, USA) and subjected to confocal laser scanning microscopy (CLSM).

Images were acquired using an Olympus FLUOVIEW FV3000 equipped with a 60× oil objective lens and a CCD camera linked to the FV3000 in-built software. Fields were selected randomly, and images were captured using Z-stacking at a resolution of 512X512 pixels. The lens had a numerical aperture of 1.42. Images of the oxidized state (stained) were captured by excitation with a 488 nm laser, and emissions were collected using a 525/50 nm filter. Post-processing and analysis of images were performed using the Olympus cellSens software. Images acquired at 96 ppi were projected in a maximized view, and for better visualization, the brightness/contrast of green fluorescence was adjusted to 1,556 from 4,095. Images were saved as “.tiff” files. The intensity profile was determined using ImageJ software. Mean intensity profiles from three independent fields at different time points were recorded and plotted using GraphPad Prism 8.0.

### Estimation of NADH/NAD+ in biofilm cells

Freshly revived *M. smegmatis* mc^2^155 transformed with the pMV762–Peredox–mCherry construct was seeded for biofilm formation, as described above, in 7H9T medium containing hygromycin (7H9H). A sterile round glass coverslip (12mm; Blue Star, India) was placed in each well of a 24-well sterile plate to allow biofilm imaging. After 2 and 4 days, the medium beneath the biofilm was carefully aspirated, and the biofilm was allowed to settle on the coverslip. The early log phase of the planktonic culture of the same strain (OD_600_ ≈ 0.3–0.4) was used as the control.

Coverslips containing biofilms were mounted on glass slides using ProLong Glass Antifade Mountant (Thermo Fisher Scientific, Oregon, USA). For controls, 100–200 µL of planktonic samples were centrifuged, and the harvested cells were mounted on glass slides using the same mounting medium. Images were acquired using a Nikon *Eclipse Ti* microscope equipped with a 60X oil immersion objective and Z-stacking capability, at a resolution of 512X512 pixels and a numerical aperture of 1.49, through a CCD camera connected to the Nikon A16 computer system running NIS-Elements software. The samples were excited with a 405 nm laser, and emission was collected using a 525/50 nm filter for T-sapphire; excitation at 561 nm and emission at 625/50 nm were used for mCherry. Images were assigned pseudo-colors to represent NADH : NAD^+^ levels in individual cells.

Post-processing and analysis were performed using Nikon NIS-Elements version 4.00.04 software, and images were saved in “.tiff” format. The relative green-to-red fluorescence intensity ratios were plotted using GraphPad Prism 8.0.

### Analysis of temporal expression of *recA* in mycobacterial biofilm

*M. smegmatis* mc²155 strains harboring the pMV262 vector with P_recA_~mClover were freshly revived on 7H10K plates. As described earlier, biofilms were seeded in 7H9 biofilm medium with a glass coverslip. After 2, 4, and 8 days of incubation, the biofilm-containing coverslips were mounted on glass slides with ProLong Glass Antifade Mountant.

Images were acquired using a Nikon Eclipse Ti microscope with the appropriate laser for mClover (λex405nm/λem525/50 nm). Post-processing and analysis were carried out using Nikon NIS-Elements version 4.00.04 software. For improved visualization, the brightness/contrast of the green channel was adjusted to 1,000 from 4,095. Images were converted to “.tiff” format, and intensity profiles were analyzed using ImageJ software. Mean intensity profiles from three independent fields at different time points were recorded and plotted using GraphPad Prism 8.0.

### Expression profiling of *recA*-dependent *dnaE2* expression in biofilms

*M. smegmatis* mc^2^155 harboring a dual reporter—pMV262 vector with *P_recA_~*mClover and *P_dnaE2_~*mCherry—was revived on a fresh 7H10K plate. Biofilms were seeded in 7H9 biofilm medium with a glass coverslip, as described earlier. After 2, 4, and 8 days of incubation, biofilm-containing coverslips were mounted on glass slides with ProLong Glass Antifade Mountant.

Images were acquired using a Nikon *Eclipse Ti* microscope equipped with a 60× oil immersion objective. Appropriate lasers were used for mClover (λ_ex_405nm/λ_em_525/50 nm) and mCherry (λ_ex_561nm/λ_em_625/50 nm) were used. Post-processing and analysis were performed using Nikon NIS-Elements version 4.00.04 software. For better image representation, the brightness/contrast of both red and green channels was adjusted to 500 from 4,095. The images were converted to “. tiff’’ format. The intensity profile was determined using ImageJ software. Mean intensity profiles from three independent fields at different time points were recorded and plotted using GraphPad Prism 8.0.

### Expression analysis of *imuA’* in biofilm

*M. smegmatis* mc²155 harboring the pMV262 vector with *P_ImuA’_~*mCherry was subjected to biofilm formation as described previously, and the expression of *ImuA’* was analyzed on the 2^nd^, 4^th,^ and 8^th^ days of incubation. Images for mCherry (λ_ex_561nm/λ_em_625/50 nm) were acquired using an Olympus FLUOVIEW FV3000 equipped with a 60X oil immersion objective lens.

Post-processing and analysis of the images were performed using the Olympus cellSens software platform. Images acquired at 96 ppi were projected in maximized view. For better image representation, the brightness/contrast for red fluorescence was adjusted to 1,250 from 4,095, and the gamma value was modified to 0.8 from 1. The images were saved as “.tiff” files. The intensity profile was determined using ImageJ software. Mean intensity profiles from three independent fields at different time points were recorded and plotted using GraphPad Prism 8.0.

### Time-course analysis of *dnaE2* expression during the biofilm recovery phase

*M. smegmatis* harboring the pMV262*P_hsp_~*mClover and *P_dnaE2_~*mCherry dual reporter strain was subjected to biofilm formation as described earlier, without a glass coverslip. After 6 days, the culture was harvested at 13,000 rpm for 1 min and washed twice with 7H9 medium containing 2% Tween 80 (7H9–high Tween). The washed bacterial pellet was resuspended in fresh 7H9K medium and incubated at 37^°^C with shaking at 175 rpm.

An aliquot of the harvested culture was concentrated and immediately mounted on a glass slide with ProLong Glass Antifade Mountant, which was considered 0h after recovery. Subsequently, samples were analyzed after 3 and 6h for expression of the dual reporters using confocal laser scanning microscopy (CLSM). Images were acquired using an Olympus FLUOVIEW FV3000 microscope with appropriate lasers for mClover (λ_ex_405nm/λ_em_525/50 nm) and mCherry (λ_ex_561nm/λ_em_625/50 nm).Fields were selected randomly and captured using Z-stacking with a 60× oil immersion objective at a resolution of 512 × 512 pixels. Post-processing and image analysis were performed using the Olympus cellSens software platform. Images acquired at 96 ppi were projected in maximized view and saved as “.tiff” files.

### Determination of bacterial cell viability by live/dead staining

Actively growing cultures of *M. smegmatis* mc^2^155 and *ΔdnaE2* strains were diluted to an OD_600_ of 0.1 in 7H9 medium and treated with ciprofloxacin (10 µg/mL) for 48h at 37^°^C with shaking.

For live/dead staining, the bacterial pellets were resuspended in 1 mL of 150 mM NaCl solution containing 0.2% Tween 80 and stained with the Live/Dead staining solution for 15 min at room temperature in the dark. The staining solution was prepared by mixing Syto9 and propidium iodide in a 1:1 ratio and reconstituting the dyes in DMSO (Invitrogen, California, USA). The samples were concentrated by mild centrifugation and mounted on glass slides with ProLong Glass Antifade Mountant, as previously described.

Images were acquired using an Olympus FLUOVIEW FV3000 confocal microscope with Z-stacking at 60× oil immersion. Syto9 (live) images were captured with λ_ex_ 405 nm/λem 525/50 nm, and PI (dead) images with λ_ex_ 561 nm/λ_em_ 625/50 nm. Post-processing and analysis of the images were performed using the Olympus cellSens software platform. For better image representation, the brightness/contrast of the green channel was adjusted to 1,556 from 4,095. The images were saved in “. tiff’’ format.

For biofilm cultures, only WT biofilms were used. After 6 days, biofilms were disrupted by repeated pipetting and processed with saline as described above. Early mid-log planktonic cultures in 7H9 with Tween were used as controls and stained with Syto9 and propidium iodide. Microscopic images were acquired and processed as described above.

The percentage survival of live bacteria was calculated by taking the ratio of green-stained cells to the total number of cells (green+red).

### Determination of mutations in biofilm and planktonic cultures of WT and *ΔdnaE2* strains of *M. smegmatis*

*M. smegmatis* mc²155 with the empty vector (WT) and *ΔdnaE2* strains were freshly revived on 7H10A plates. The cultures were adjusted to an OD_600_ of ~2.0, and 20 µL of each was added to 2 mL of 7H9 biofilm medium in sterile flat-bottom 24-well plates for biofilm culture and to culture tubes containing 7H9T medium for planktonic culture. The starting OD_600_ for both systems was ~0.02. For each strain, 12 replicates were maintained for both planktonic and biofilm cultures.

The biofilm cultures were incubated at 30°C under static conditions, while the planktonic cultures were incubated at 37°C in a shaker incubator at 175 rpm. Once the planktonic cultures reached an OD_600_ of ~0.4–0.6, 50 µL aliquots from each replicate were subjected to tenfold serial dilution up to 10^-4^ to enumerate viable counts. The remaining culture was harvested in sterile 2 mL microcentrifuge tubes at 13,000 rpm for 1 min at room temperature. The supernatant was discarded, and the pellet was resuspended in 0.1 mL of 7H9 medium. The suspension was spread on 7H10 plates containing 2.5 µg/mL ciprofloxacin (Cip).

For biofilms, cultures on the 4th and 6th days were disrupted, and cells were harvested. The harvested cells were washed twice with 7H9 medium containing high Tween 80. The same procedure used for planktonic cultures was followed to enumerate viable and mutant counts in the biofilm samples.

For biofilm recovery samples, 10 replicates of 2 mL biofilm cultures were disrupted, washed twice with high-Tween 7H9, and incubated in 7H9 (with Tween) for 3h for recovery. Plates were incubated at 37°C; viable counts were recorded after 3 days, and Cip^R^ mutants were recorded after 5 days.

The mutation frequency was calculated by dividing the number of mutants obtained on the Cip plates by the corresponding viable count. Statistical analysis and graphing were performed using GraphPad Prism version 8.0.

### Competition assay in biofilm

*M. smegmatis* mc^2^155 with *P_hsp_*~mClover (WT) and *ΔdnaE2 with P_hsp_*~mCherry reporter strains were freshly revived on 7H10K plates. For each strain, a bacterial suspension with an OD_600_ of ~2.0 was prepared.

The competition experiment was initiated by mixing the competing strains—WT (*P*_hsp_~mClover) versus *ΔdnaE2* (*P*_hsp_~mCherry)—in a 1:1 ratio, with each adjusted to an OD_600_ of ~0.02. The final cell density in the biofilm corresponded to an OD_600_ of approximately 0.02 in 2 mL of 7H9K biofilm medium in a sterile 24-well flat-bottom plate. This 1:1 mixture was considered the “0^th^ passage.”

The cultures were incubated for 6 days at 30°C under static conditions to obtain the “1^st^ passage.” From this, 100–200 µL of a homogeneous bacterial suspension was plated on 7H10K agar. Biofilms were reseeded with bacterial inocula corresponding to an OD_600_ of ~0.02. The seeding and subsequent subculturing process were repeated until the 6^th^ passage was obtained.

Quadrant streaking was performed at the 0^th^ and 6^th^ passages on 7H10K plates to obtain isolated colonies, which were patched on 7H10HK and 7H10K media to enumerate *ΔdnaE2* and WT colonies, respectively. At the end of every passage, 100–200 µL aliquots of the culture were collected and processed for microscopic analysis.

Images were acquired using a Nikon *Eclipse Ti* microscope with appropriate lasers for mClover (λ_ex_405nm/λ_em_525/50 nm) and mCherry (λ_ex_561nm/λ_em_625/50 nm). Post-processing and analysis were performed using Nikon NIS-Elements version 4.00.04 software. A 3× zoom was applied in the area of focus for better representation. The images were converted to “.tiff” format.

### RNA isolation and sequencing of the biofilm and the planktonic cultures

*M. smegmatis* mc²155 strain was freshly revived on a 7H10 plate. As described earlier, biofilm and planktonic cultures were seeded in 7H9 biofilm medium and 7H9 medium containing Tween 80, respectively. For biofilm recovery samples, the biofilm was disrupted, washed twice with 7H9 medium containing high Tween 80, and incubated in 7H9 (with Tween) for 3h. Cells were harvested after the respective incubation periods.

The harvested pellets from each condition were immediately frozen in liquid nitrogen and stored at −80 °C. The pellets were resuspended in 1 mL of TRI reagent by gentle pipetting on ice. This suspension was transferred into sterile 2 mL bead-beating tubes containing 0.5mm zirconia–silica beads (Biospec, USA). Bead beating was performed using a Mini-Beadbeater (BioSpec, USA) in two cycles of 1 min each, with intermittent cooling on ice for 2 min between cycles.

The tubes were centrifuged at 13,000 rpm for 10 min at 4°C in an Eppendorf 5804 R centrifuge with a microcentrifuge rotor. The supernatant from the lysate was transferred to a new sterile tube, leaving behind the cell debris and beads. Total RNA was extracted and treated with 2U of RNase-free DNase according to a previously described protocol (Kurthkoti et al., 2017), excluding the RNA coprecipitation agent. RNA was further purified using an RNA isolation kit (Macherey–Nagel, Germany) following the manufacturer’s protocol. RNA quality was assessed by UV spectrophotometry and agarose gel electrophoresis.

### Library preparation

Briefly, 1 µg of total RNA from each sample (three groups, with duplicate samples in each group) was depleted of bacterial rRNA using the NEBNext rRNA Depletion Kit (Cat. No. E7850X; New England Biolabs, Ipswich, MA, USA) according to the manufacturer’s protocol, which included a DNase I digestion step. The rRNA-depleted RNA was purified using RNA purification beads, as instructed.

The NEBNext Ultra II Directional RNA Library Prep Kit for Illumina (Cat. No. E7760L) was used to construct double-stranded cDNA libraries from rRNA-depleted RNA. Library preparation involved RNA fragmentation, first-strand cDNA synthesis with random primers, second-strand cDNA synthesis, end repair of double-stranded cDNA, adapter ligation, removal of excess adapters using sample purification beads, PCR enrichment of adapter-ligated DNA, and cleanup of PCR products, as instructed by the manufacturer.

The cleaned libraries were quantified using a Qubit fluorometer (Thermo Fisher Scientific, Waltham, MA, USA), and appropriate dilutions were loaded onto a TapeStation 4200 High Sensitivity D1000 ScreenTape (Agilent Technologies, Santa Clara, CA, USA) to determine the fragment size range and average library size.

### Sequencing and data processing

Libraries were diluted to 4 nM, pooled, spiked with 5% PhiX pre-made library (Illumina, San Diego, CA, USA), and loaded onto a MiSeq v3 kit (Illumina). Sequencing was performed for 1X150 cycles. The original raw data from the Illumina MiSeq were transformed into sequence reads by base calling.

Raw data were recorded in FASTQ files, and reads per sample ranged from 3,497,155 to 5,051,470. Read quality was assessed using *FastQC* v0*.11.3* (Andrews, 2012) before downstream analysis. Low-quality reads were trimmed using *Fastpv 0.20.1–* (Chen et al., 2018) when base quality dropped below 30, and adapters were removed. The reads were rechecked for quality improvement before proceeding.

A transcriptome index was prepared using the cDNA reference assembly of *M. smegmatis* mc^2^155 obtained from NCBI (https://www.ncbi.nlm.nih.gov/assembly/GCF_000015005.1/). The quality passed reads were mapped onto the reference transcriptome and quantified using *Kallisto v0.46.2* (Bray et al., 2016). The aligned reads ranged from 85.39% to 92.35%.

### Bioinformatic analysis for RNA sequencing

The *Degust v4.1.1 web tool* (David, 2019) was used for differential expression analysis. Only genes with counts per million (CPM) ≥ 1 were analyzed further. Genes were filtered based on a false discovery rate (FDR) ≤ 0.05 and a minimum fold change (FC) ≥ 2.

The sequence reads have been deposited in the SRA database under the project accession PRJNA888550 (RNA-Seq of *Mycobacterium* from planktonic, biofilm, and recovery samples).

### Genomic DNA isolation and sequencing from biofilm cultures

*M. smegmatis* mc²155 was seeded for biofilm formation in triplicate as described earlier and maintained for three passages. Genomic DNA was isolated directly from the triplicate biofilm cultures (Salini et al., 2022). As a control, genomic DNA from the same strain was isolated from three independent replicates of planktonic cultures in the early stationary phase (OD_600_ 1-1.2).

### Library preparation and QC

The whole-genome sequencing (WGS) library was prepared using the QIAseq FX DNA Library Kit for Illumina (Cat. No. 180479; QIAGEN) with an input of 300 ng of DNA per sample. Library concentrations were determined using a Qubit 3.0 Fluorometer (Cat. No. Q33216; Life Technologies) and the Qubit dsDNA HS (High Sensitivity) Assay Kit (Cat. No. Q32854; Thermo Fisher Scientific). Library quality was assessed using the Agilent D5000 ScreenTape System on a 4150 TapeStation (Cat. No. G2992AA; Agilent).

### Whole genome sequence analysis

Sequencing was performed at 200× depth. Sequence reads were quality-filtered using Trimmomatic software (Bolger et al., 2014) to remove *E. coli* and adapter contamination. Quality filtering was performed using a sliding window of 4 bp; sequences with an average Phred score <15 were clipped. Trimmomatic QC parameters were set as follows: leading:3, slidingwindow:4:15, trailing:3, minlen:60. Reads shorter than 60 bp were discarded.

Filtered SE/PE reads were mapped onto the *M. smegmatis* mc²155 reference genome (accession number GCF_013349145.1) using the Burrows–Wheeler Aligner (BWA-MEM) algorithm (Li and Durbin, 2009). The Genome Analysis Toolkit (GATK) pipeline (Auwera et al., 2013) was used for sorting, PCR duplicate removal, and realignment. All predicted gVCF files were merged, and variants were identified using the GATK pipeline.

SNPs with a read depth <5 or mapping quality <20 were excluded. SNPs with missing calls in >20% of accessions were removed. Annotation of filtered SNPs was performed using SNPEff software (Cingolani et al., 2012). Circos plots were generated to visualize SNPs under different conditions using the Python pycircos module.

The sequence reads have been deposited in the SRA database under the project accession PRJNA946277 (Whole-genome sequencing of planktonic and biofilm cultures).

### Biofilm stability assay

Bacterial biofilms of *WT*, Δ*dnaE2*, and complemented strains were seeded for biofilm formation in 24-well plates containing 7H9 medium without Tween 80. Biofilm images were acquired on the 6^th^ and 21^st^ days, and the presence of pellicles was recorded.

### Isolation of eDNA from biofilm

Biofilm cultures were set up as described in the Methods section. Cultures from the 2^nd^, 4^th^, and 6^th^ days (three replicates per time point) were harvested by centrifugation at 11,000 × g for 1 min. The supernatants were collected and filtered through 0.22 µm filters (Sartorius Minisart). The filtrates were transferred to new tubes, and an equal volume of phenol:chloroform:amyl alcohol (25:24:1) was added. The samples were mixed on a mini-rotator for 5min and centrifuged at 7,500 rpm for 5min.

The supernatant was carefully collected and transferred to sterile 15 mL Falcon tubes. To each supernatant, one-tenth volume of 3 M sodium acetate was added and mixed gently. eDNA was precipitated by adding an equal volume of ice-cold isopropanol and incubating the mixture at −20°C. After incubation, the samples were centrifuged at 10,000 rpm for 15 min at 4°C. The supernatants were decanted, and pellets were washed twice with 2 mL of 75% ethanol at 10,000 rpm for 7 min at 4°C.

The tubes were air-dried, and the pellets were resuspended in 50 µL of autoclaved water and mixed gently. The eDNA samples were run on 1% agarose gels in 1× TAE buffer. Black triangles indicate eDNA isolated from biofilm cultures at the corresponding time points (2 days, 4 days, and 6 days). A representative image is shown.

## Data Availability

The datasets presented in this study can be found in online repositories. The names of the repository/repositories and accession number(s) can be found in the article/**Supplementary Material**.
